# Ag-BN/HNT-TiO_2_ nanofibers produced by electrospinning as catalysts to remove acetaminophen

**DOI:** 10.1016/j.heliyon.2024.e24740

**Published:** 2024-01-17

**Authors:** Mahmoud Abid, Igor Iatsunskyi, Emerson Coy, Geoffroy Lesage, Abdesslem Ben Haj Amara, Mikhael Bechelany

**Affiliations:** a Institut Européen des Membranes, IEM, UMR 5635, Univ Montpellier, ENSCM, CNRS, 34730, Montpellier, France; b Laboratory of Resources, Materials & Ecosystem (RME), University of Carthage, Faculty of Sciences of Bizerte, Zarzouna, 7021, Tunisia; c CNRS, Grenoble INP, LMGP, Institute of Engineering, Université Grenoble Alpes, 38000, Grenoble, France; d NanoBioMedical Centre, Adam Mickiewicz University, Wszechnicy Piastowskiej 3, 61-614, Poznan, Poland; e Gulf University for Science and Technology, GUST, Kuwait

**Keywords:** Nanofibers, Photocatalysis, Acetaminophen, Boron nitride, Silver nanoparticles, Halloysite nanotubes

## Abstract

In this study, we present a novel approach to enhancing the degradation of acetaminophen (ACT) using nanostructured hybrid nanofibers. The hybrid nanofibers were produced by employing both sol-gel and electrospinning methodologies, integrating precise quantities of silver (Ag) and boron nitride (BN) nanosheets into titanium oxide (TiO_2_) nanofibers and halloysite nanotubes (HNT). We extensively examined the morphology, structure, and optical properties of these materials by employing scanning electron microscopy, X-ray diffraction, Raman spectroscopy, and X-ray photoelectron spectroscopy in our analysis. In the case of the HNT-TiO_2_ composite, the introduction of Ag nanoparticles at concentrations of 0.5%, 1.5%, and 3% led to a significant improvement in photocatalytic activity. Under visible light exposure for 4 h, the photocatalytic activity increased from 63% (HNT-TiO_2_) to 78.92%, 91.21%, and 92.90%, respectively. This enhancement can be attributed to the role of Ag nanoparticles as co-catalysts, facilitating the separation of electrons and holes generated during the photocatalytic process. Furthermore, BN nanosheets served as co-catalysts, capitalizing on their distinct attributes, including exceptional thermal conductivity, chemical stability, and electrical insulation. The incorporation of BN nanosheets into the Ag (3%)/HNT-TiO_2_ composite at a concentration of 5% resulted in a remarkable increase in ACT degradation efficiency. The degradation efficiency improved from 59.47% to an impressive 99.29% within a 2-h irradiation period due to the presence of BN nanosheets. Toxicity and scavenging assays revealed that OH^•−^, O_2_^•−^, and h^+^ were the major contributors to ACT degradation. Moreover, across five consecutive cycles, the Ag-BN/HNT-TiO_2_ composite exhibited consistent and stable performance, underscoring the significant contributions of Ag and BN in augmenting the photocatalytic capabilities of the composite. Overall, our findings suggest that this novel hybrid nanofiber composite holds great promise for practical applications in environmental remediation due to its improved photocatalytic activity and stability.

## 1 Introduction

Due to rapid population growth, industrialization, and global development, human activities have significantly expanded, leading to increased chemical utilization [1,2]. Consequently, pharmaceuticals, insecticides, dyes, and personal care products have contaminated aquatic resources, emerging as a novel class of contaminants [[3], [4], [5]]. Discharging untreated water containing these micropollutants into the environment has led to water pollution [6]. Even at low concentrations, emerging contaminants can have detrimental effects on human health and profound impacts on ecosystems [[7], [8], [9]]. Thus, the development of efficient purification systems to eliminate these target pollutants has become a top priority, especially in the context of current water scarcity [[10], [11], [12]].

In recent years, two primary strategies have been explored to address the removal of these chemical contaminants from water sources [6,13]. The first approach involves the adsorption of pollutants onto solid materials, while the second method focuses on their mineralization. Adsorption on solid sorbents remains the most commonly employed method for effective purification [14]. However, this approach has its primary drawback: the transmission of organic contaminants to the sorbent, making regeneration challenging and costly [15]. As a result, this process falls short of meeting the current demand for an energy-efficient and cost-effective treatment method. On the other hand, advanced oxidation processes, particularly heterogeneous photocatalysis [16,17], offer an environmentally friendly and energy-efficient solution for breaking down and mineralizing non-biodegradable compounds found in wastewater treatment [[18], [19], [20]]. Photocatalysis depends on semiconductors that can absorb photons possessing energies that match or surpass the bandgap of the semiconductor material. This process generates charge carriers with oxidative or reductive potential, accelerating chemical reactions and facilitating the degradation of pollutants through the generation of reactive species [21].

Titanium dioxide (TiO_2_), particularly in its anatase form, is considered a highly intriguing photocatalyst due to its exceptional activity, stability, and cost-effectiveness [22,23]. However, the wide bandgap of anatase TiO_2_ limits its absorption to a small fraction of sunlight (approximately 3%–5%), primarily in the UV radiation range. Additionally, anatase TiO_2_ exhibits limited recovery capacity and rapid electron-hole recombination [24,25]. Thus, there is a pressing need to develop economically viable photocatalysts that can be activated by visible-spectrum radiation [26]. Recent research efforts have successfully immobilized TiO_2_ on inert supports such as glass tubes, fibers, and stainless steel. However, the efficiency of this system has been compromised due to a reduction in surface area [27,28]. Numerous studies have also explored porous materials as adsorption supports for pollutants, including silica gel, zeolites, and clays. Among these materials, fibrous minerals like palygorskite, sepiolite, and halloysite are particularly intriguing due to their capacity to absorb and retain pollutants. Moreover, these minerals can be structurally modified through cation exchange characteristics and the presence of silanol groups [29]. Notably, several studies have demonstrated that using halloysite (HNT) as a support material prevents TiO_2_ agglomeration and facilitates the development of adsorbents and catalysts with larger specific surface areas, an essential element in improving processes for removing pollutants [30,31]. Furthermore, our group has showcased the potential of the TiO_2_ and HNT combination, represented by HNT-TiO_2_ nanofibers, for effective pollutant reduction through photocatalysis [32,33].

In parallel, many researchers have explored strategies to reduce the fast electron-hole recombination of TiO_2_ by incorporating various promoters, such as palladium (Pd) [34], silver (Ag) [35,36], zinc (Zn) [37], ferrous metals or oxides [[37], [38], [39], [40]] , boron nitride (BN) and platinum (Pt) [34,41]. These enhancers are crucial in bolstering the separation of charges and minimizing recombination, consequently enhancing the overall photocatalytic efficacy. Of particular interest is boron nitride (BN), characterized by its expansive surface area and wide band gap (>5.5 eV), along with its chemical stability and semiconducting properties, which resemble metals and metal oxides [33,35,38]. Our prior research has revealed that the presence of BN nanosheets significantly enhances the photocatalytic performance of HNT- TiO_2_ nanofibers under visible light exposure. This notable effect can be largely attributed to efficient charge separation, facilitated by the electrostatic interaction between the BN layer and TiO_2_, promoting the movement of charge carriers from the bulk TiO_2_ toward its surface, ultimately enhancing pollutant degradation under visible light exposure [33].

To further enhance the photocatalytic properties of HNT-TiO_2_ nanofibers, we have produced Ag/HNT-TiO_2_ and BN-Ag/HNT-TiO_2_ composite nanofibers by electrospinning. The exfoliation of BN sheets was achieved through a process involving high-power sonication and centrifugation, with pork gelatin dissolved in hot water. The Synthesis of silver nanoparticles involved dissolving AgNO_3_ in acetic acid, followed by a comprehensive assessment of the nanoparticles' structure and morphology. Subsequently, we conducted a series of experiments to evaluate the photocatalytic effectiveness of Ag/HNT-TiO_2_ and BN-Ag/HNT-TiO_2_ composite nanofibers under visible light and employed high-performance liquid chromatography (HPLC) to identify reaction intermediates and by-products. Additionally, toxicity screening tests and scavenger tests were conducted to evaluate the acute toxicity of by-products formed during acetaminophen (ACT) degradation and to determine the reactive species implicated in ACT degradation, respectively.

## 2 Materials and methods

### 2.1 Materials

Table 1 describes the chemicals used (without any further purification) in this study.

**Table 1:** Materials used in this study.

Material	Supplier/Source	Purity	CAS number
Halloysite (HNT)	Tamra (Nefza District, NW Tunisia)	–	–
Titanium tetraisopropoxide (TTIP)	Sigma–Aldrich	97%	546-68-9
Polyvinyl pyrrolidone (PVP)	Sigma–Aldrich	Mw = 1,300,000	9003-39-8
Sodium chloride	Sigma–Aldrich	≥99%	7647-14-5
gelatine from porcine skin	Sigma–Aldrich	–	9000-70-8
Silver nitrate	Sigma–Aldrich	≥99%	7761-88-8
Acetaminophen (ACT)	Sigma–Aldrich	≥99%	103-90-2
2-propanol (IPA)	Sigma–Aldrich	99.9%,	67-63-0
*p*-benzoquinone (BQ)	Sigma–Aldrich	≥99.5%,	106-51-4
Ethylenediaminetetraacetic acid (EDTA)	Sigma–Aldrich	99.995%,	60-00-4
Acetic acid	VWR chemicals	–	64-19-7
Nafion perfluorinated resin	Sigma–Aldrich	–	31175-20-9
Ethanol	VWR chemicals	≥99.8%,	64-17-5
Deionized water	Milli-Q® Academic	>18.2 MΩ	

### 2.2 Fabrication of Ag/HNT-TiO_2_ and BN-Ag/HNT-TiO_2_ composite nanofibers

Electrospinning was employed to create Ag/HNT-TiO_2_ and BN-Ag/HNT-TiO_2_ nanocomposites with varying Ag and BN content, determined based on HNT-TiO_2_ weight percentage (wt%). The method for preparing the spinning solution closely followed the procedures utilized in our previous studies [32,33,39,42,43]. Initially, two distinct solutions, referred to as Solutions A and B were meticulously prepared as follows. In the case of the first solution, Solution A, a mixture of natural halloysite nanotubes (HNT) and titanium tetraisopropoxide (TTIP) in a weight ratio of 95:5, along with exfoliated boron nitride (comprising 5 wt% of the HNT-TiO_2_ mass), was added to a solvent blend comprising 2 mL of ethanol and 2 mL of acetic acid. The resulting blend underwent a thorough process of sonication and stirring, lasting for a duration of 3 h. This meticulous procedure yielded a uniformly mixed Solution A, which was subsequently prepared for use. For Solution B, the preparation involved the dissolution of AgNO_3_ (constituting 0.5, 1.5 or 3 wt% of HNT-TiO_2_ mass), within 2 mL of acetic acid. The dissolution process required 12 h of sonication to ensure the formation of a stable AgNO_3_ solution. Subsequently, Solutions A and B were combined and further subjected to 2 h of sonication to achieve the necessary viscosity for electrospinning. Additionally, a polymeric solution, denoted as Solution C, containing 0.3 g of PVP and 3 mL of ethanol, was added to the mixture, and the entire mixture was stirred at room temperature overnight to achieve the required viscosity for the electrospinning process. This process entailed injecting the solution into a 20 mL plastic syringe, which was linked to a high-voltage source through a stainless steel needle. Specific electrospinning parameters were applied: the needle tip was positioned at a 10 cm distance from the collector, a voltage of 25 kV was applied, and the solution was administered steadily at a pace of 1 mL per hour. The resulting nanofibers were left to undergo hydrolysis in air overnight and subsequently heated at 400 °C in an air environment for 4 h. Details regarding the nanofibers used in this investigation are provided in Table 2.

**Table 2:** Ag/HNT-TiO_2_ and BN-Ag/HNT-TiO_2_ samples used in this study.

Sample name	Sample description	BN content (%)	Ag content (%)
Ag 0.5	Ag 0.5/H95T5	0	0.5
Ag 1.5	Ag 1.5/H95T5	0	1.5
Ag 3	Ag 3/H95T5	0	3
AgHBN	Ag 3/BN5/H95T5	5	3

H95T5: 95% HNT and 5% TiO_2_.

### 2.3 Structural characterizations

The comprehensive analysis of the composite nanofiber samples encompassed various techniques, such as scanning electron microscopy (SEM), transmission electron microscopy (TEM), Brunauer-Emmett-Teller (BET) analysis for surface area determination, X-ray diffraction (XRD) for crystal structure examination, Raman spectroscopy for molecular insights, and X-ray photoelectron spectroscopy (XPS) to determine elemental composition and chemical states. These methods have been previously outlined in our earlier research endeavors [32,33,43].

### 2.4 Photocatalytic and micro-toxicity tests

The evaluation of ACT degradation efficiency under visible light and micro-toxicity tests using the bioluminescent marine bacterium *Vibrio fischeri* were conducted as described in our previous reports [32,33,43].

## 3 Results and discussion

### 3.1 Morphological and structural analysis of Ag/HNT-TiO_2_ and BN-Ag/HNT-TiO_2_ nanocomposites

Nanocomposites were fabricated using the electrospinning technique, incorporating increasing amounts of Ag within TiO_2_ nanofibers. After annealing at 400 °C in an air environment for 4 h, these samples exhibited a subtle grayish color. SEM analysis of the Ag/HNT-TiO_2_ and BN-Ag/HNT-TiO_2_ composite nanofibers revealed their nanofibrous morphology.

**Fig. 1:**
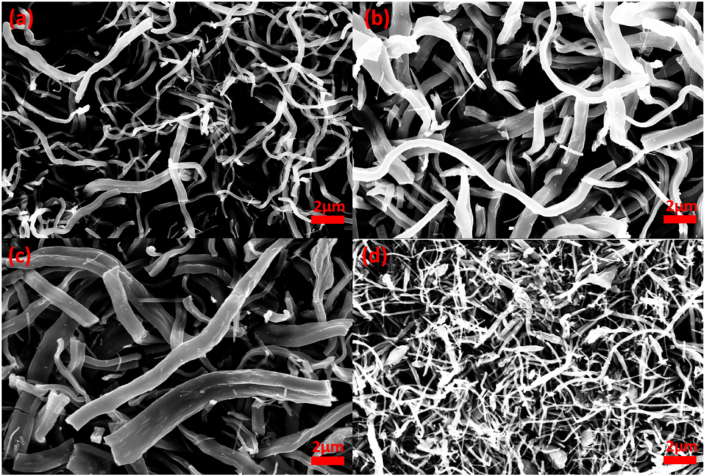
Scanning electron microscopy photographs of the Ag-0.5 (a), Ag-1.5 (b), Ag-3 (c), and AgHBN (d) samples (described in Table 2).

The morphological analysis by SEM of the Ag-0.5, Ag-1.5, and Ag-3 samples (Table 2) showed that in all three samples, nanofibers were uniform, continuous, and oriented randomly (Fig. 1a–c). Although all samples were fabricated using the same electrospinning conditions and based on our previous work [32,33], the mean nanofiber diameter slightly increased from 233 ± 10 nm (H95T5: nanocomposite with 95% HNT and 5% TiO_2_ and without Ag and BN) to 256 (Ag-0.5), 368 (Ag-1.5) and 396 ± 10 nm (Ag-3). This observation aligns with our previous findings [32,33], which establish a significant relationship between fiber diameter and dopant content. This could be explained by AgNO_3_ addition that increased the total metal content and viscosity of the spinning solution to be used for electrospinning. The Ag-3 sample was chosen on the basis of the specific requirements for the intended application and of the nanofiber mechanical and surface properties. However, AgHBN nanofibers (Ag 3% and BN; Table 2) were not uniform in size (Fig. 1d), and two size ranges were observed, possibly due to the agglomeration of BN nanosheets and Ag nanoparticles. This observation strongly supports the existence of BN nanosheets in the AgHBN samples [32,33].

**Fig. 2:**
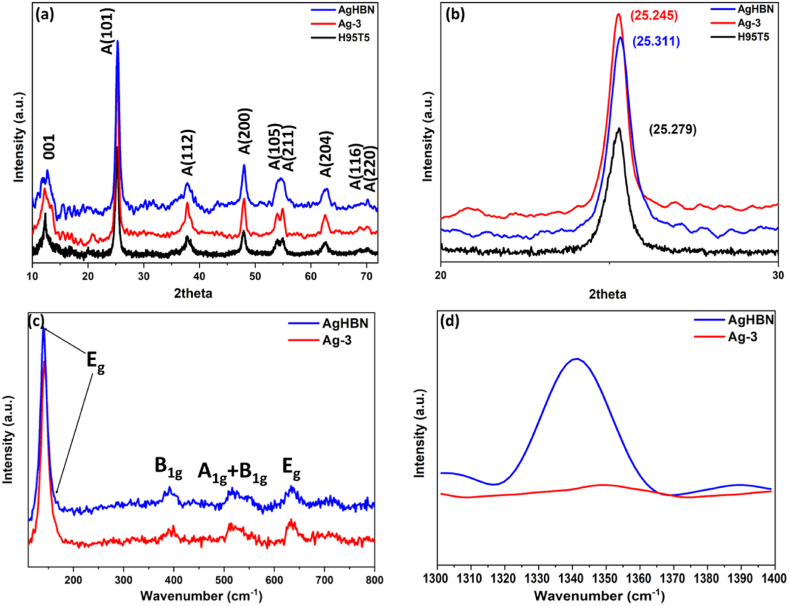
XRD patterns (a,b) and Raman spectra (c,d) of composite nanofibers with Ag-3 and AgHBN.

Distinctive peaks were observed at specific 2θ values in the XRD patterns of the H95T5, Ag-3, and AgHBN samples, as illustrated in Fig. 2a and b. The observed peaks aligned with the 001 reflection of HNT (measuring at 7.18 Å) and the anatase phase of TiO_2_. The tetragonal arrangement exhibited reflections at 2θ values of 25.31, 37.82, 48.05, 54.52, 54.50, 62.50, 68.93, and 70.16°, corresponding to the (101), (112), (200), (105), (211), (204), (116), (220), and (215) Miller planes, respectively, as previously documented [32].

The XRD analysis revealed that Ag and BN addition induced shifts in the diffraction peaks of the TiO_2_ nanofibers, indicating changes in their crystal structure.

With the inclusion of Ag, the diffraction peaks of Ag/TiO_2_ composite nanofibers were observed at lower 2θ values than those of H95T5 nanofibers. The noted alteration (Fig. 2b) is likely due to the integration of Ag into the TiO_2_ lattice, causing distortion owing to differences in ionic radius and crystal structures between TiO_2_ and Ag. Consequently, this lattice distortion results in an increase in d-spacing and a decrease in diffraction peak intensity. In line with our findings, previous studies have also demonstrated that increasing Ag content leads to a downward shift of the TiO_2_ (101) peak to lower angles [44,45].

Conversely, upon the BN addition, the TiO_2_ diffraction peak shifted to higher 2θ values. This corresponds to an expansion of the d-spacing with the inclusion of 5 wt % BN in TiO_2_. This shift can be attributed to the creation of a new TiO_2_-BN phase characterized by smaller lattice parameters and shorter d-spacing compared to pure TiO_2_. This is due to the fact that BN has a hexagonal crystal structure and TiO_2_ has a tetragonal crystal structure. The lattice mismatch between these phases leads to a distortion in the TiO_2_ lattice, resulting in a decreased d-spacing and a diffraction peak shift [38].

Six distinguishable Raman active modes (A1g, 2B1g, 3 Eg) were identified and attributed to the pure anatase phase of TiO_2_, as illustrated in Fig. 2c. The Raman spectra of AgHBN samples included a peak for BN at ∼1341 cm^−1^, referring to the E2g mode of BN (Fig. 2d). The addition of Boron nitride to the sample resulted in an increased intensity of this peak, confirming the incorporation of BN in the AgHBN nanocomposites [33].

**Fig. 3:**
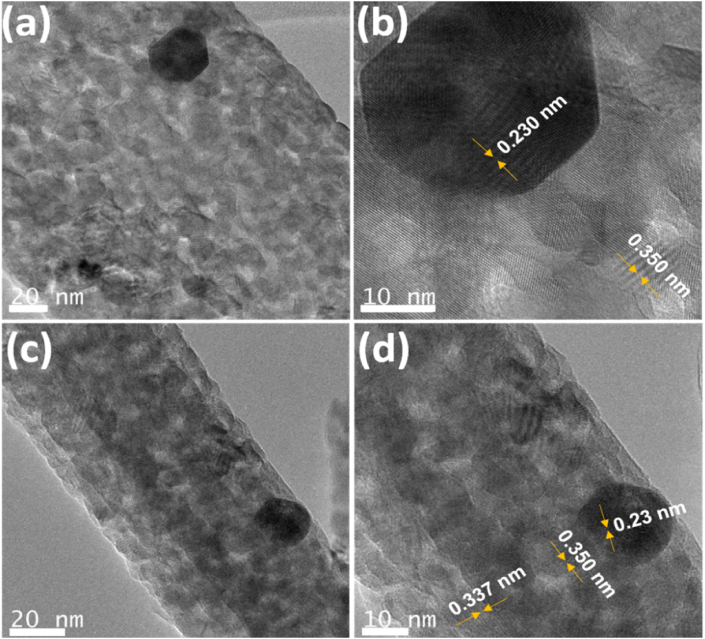
Transmission electron microscopy images of Ag-3 (a,b) and AgHBN (c,d).

Detailed examination using high-resolution TEM revealed striking similarities in the surfaces of Ag-3 and AgHBN (Fig. 3a–d), both exhibiting rough and extensive areas with lattice spacing of 0.350 nm and 0.230 nm, respectively, consistent with the (101) crystalline plane of anatase TiO_2_ and Ag (111) distances [33,46]. The AgHBN sample displayed a lattice spacing of 0.337 nm, aligning with the BN (002) plane as documented in Ref. [33].

**Fig. 4:**
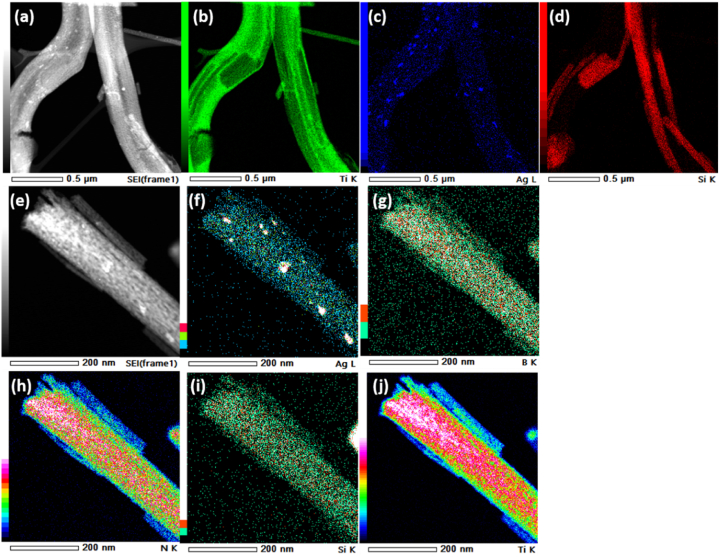
Scanning transmission electron microscopy in Conjunction with Energy Dispersive X-ray Spectroscopy for Elemental Mapping of Ag-3 (a–d) and AgHBN (e–j) Samples.

Elemental mapping showed the homogenous distribution of Ti, Si and Ag in the Ag-3 sample (Fig. 4a–d) and the distribution of Ti, Si, Ag, B and N elements in the AgHBN sample (Fig. 4e–j). Scanning TEM demonstrated that BN addition increased TiO_2_ nanofiber dispersion and Ag nanoparticle uniform distribution in the nanofiber matrix due to their synergistic effect [44].

The specific surface areas of H95T5, Ag-3 and AgHBN, calculated with the BET method, were 39.90, 63.57 and 71.70 m^2^/g, respectively. The observed expansion in lattice spacing can be attributed to the integration of Ag nanoparticles and BN nanosheets within the structure of TiO_2_ nanofibers. The inclusion is anticipated to elevate the photocatalytic efficacy by generating novel active adsorption sites [45].

**Fig. 5:**
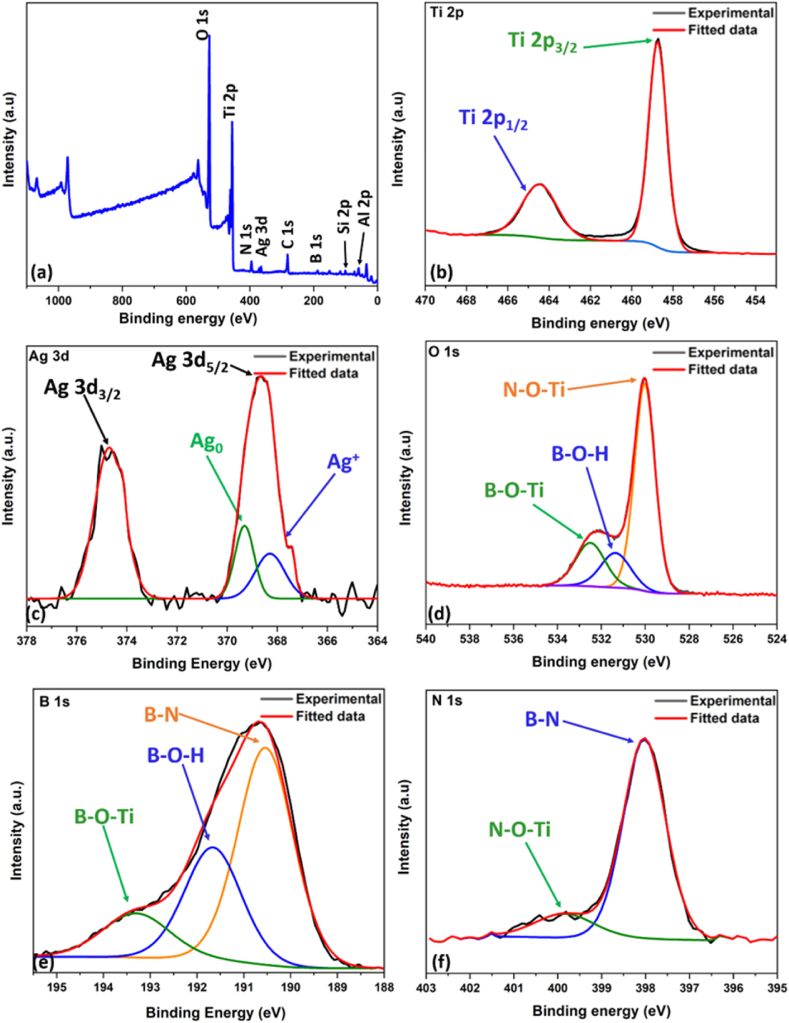
Chemical State of AgHBN via X-ray Photoelectron Spectroscopy: (a) overall survey, (b) Ti 2p, (c) Ag 3d, (d) O 1s, (e) B 1s, and (f) N 1s spectra.

The surface structure and chemical state of each element in the AgHBN sample were then studied by XPS, revealing distinct signal intensity (Fig. 5a). Strong signals were observed for Ti 2p and O 1s, while weaker signals were detected for C 1s, Al 2p, Si 2p, Ag 3d, N 1s, and B 1s.

Within the Ti 2p spectrum (Fig. 5b), two peaks were evident at 458.71 and 464.51 eV, corresponding to Ti 2p_3/2_ and Ti 2p_1/2_ states, characteristic of Ti^4+^ [33,47]. Likewise, the Ag 3d spectrum (Fig. 5c) exhibited twin peaks at 368.67 and 374.75 eV, representing Ag 3d_5/2_ and Ag 3d_3/2_ states, respectively. The shift of the binding energy to a lower value in the Ag 3d_5/2_ peak indicated the chemical reduction of Ag ions to Ag metal [[48], [49], [50]].

The Ag 3d_5/2_ peak exhibited additional deconvolution into two component peaks at 368.35 and 369.31 eV, representing different Ag states: metallic (Ag^0^) and ionic (Ag^+^) [[48], [49], [50]].

Within the O 1s spectrum, three peaks at 530.06, 531.3, and 532.7 eV were identified, corresponding to Ti–O–N, B–O–H, and B–O–Ti interactions (Fig. 5d). In the B 1s spectrum (Fig. 5e), peaks at 190.60, 191.71 and 193.42 eV were assigned to B–N, B–O–H, and B–O–Ti, respectively. Deconvolution of the N 1s peaks (Fig. 5f) showed the presence of two peaks at 398.01 and 399.81 eV, corresponding to B–N and N–O–Ti, consistent with observations in the O1 s and B 1s spectra [33]. This analysis provided conclusive evidence of BN incorporation into the TiO_2_ lattice, indicating its uniform dispersion throughout the material.

### 3.2 Photocatalytic investigations

**Fig. 6:**
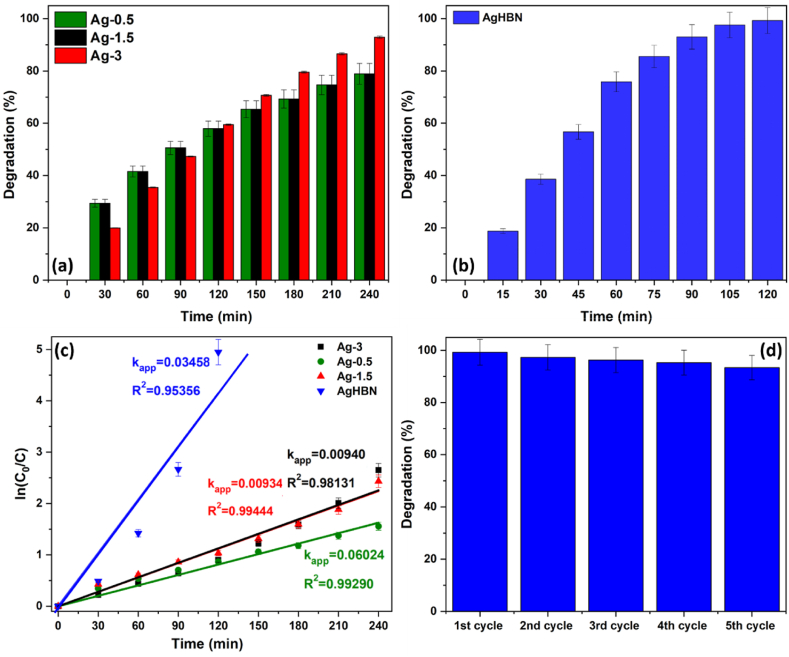
Acetaminophen (ACT) photocatalytic degradation: Comparing Ag-0.5, Ag-1.5, or Ag-3 under visible light (a), AgHBN (b), kinetics of ACT degradation in the presence of the indicated nanocomposites (c), and ACT degradation of AgHBN for five cycles (d).

The evaluation of photocatalytic performance for the Ag-0.5, Ag-1.5, and Ag-3 samples involved observing the degradation of ACT during a 4-h exposure to visible light. ACT was chosen due to its global consumption and prevalence in natural water and wastewater samples. ACT is known to be challenging to photodegrade without a catalyst [32]. Following a 4-h duration, it was noted that the degradation rate of ACT exhibited a direct correlation with the Ag content in the samples: 63% for H95T5, 78.92% for Ag-0.5, 91.21% for Ag-1.5, and 92.90% for Ag-3. (Fig. 6a). This trend can be attributed to the deposition of Ag species on the TiO_2_ nanofiber surface, leading to the capture of electrons and holes generated during the photocatalytic process. Additionally, these electrons demonstrate effective transfer to the oxygen molecules adsorbed on the TiO_2_ surface [51,52].

Furthermore, when doped with BN, the ACT degradation rate significantly increased, reaching 99.29% for AgHBN compared to 59.47% for Ag-3 after 2 h of visible light exposure (Fig. 6b). The improved performance is attributed to BN doping, facilitating the segregation of electron-hole pairs owing to electrostatic interactions. This effect can be attributed to the promotion of electron transfer from activated TiO_2_ to the negatively charged surface of BN nanosheets [33,53]. The incorporation of BN in the composite increases the availability of photoelectrons, contributing to the improved photodegradation efficiency compared to Ag-3 and previously reported photocatalysts (Table 3).

**Table 3:** AgHBN, Ag-3, and other photocatalysts (previous studies) used to degrade water pollutants.

Pollutant (mg/L)	Photocatalyst (g/L)	Synthesis method	Irradiation type	Removal efficiency (%)	Degradation time (min)	Ref.
Acetaminophen (ACT) (10 mg/L)	AgHBN (0.5 g/L)	Sol-gel + electrospinning	Halogen linear lamp	99	120	This work
ACT (10 mg/L)	Ag-3 (0.5 g/L)	Sol-gel + electrospinning	Halogen linear lamp	93	240	This work
ACT (10 mg/L)	BN5 (0.5 g/L)	Sol-gel + electrospinning	Halogen linear lamp	99	180	[33]
ACT (10 mg/L)	H95T5 (0.5 g/L)	Sol-gel + electrospinning	Halogen linear lamp	91	360	[32]
Methylene blue (MB) (20 ppm)	BN5–Ag_3_/TiO_2_ (0.4 g/L)	Sol-gel + electrospinning	Visible light (150 W)	98	80	[35]
MB (100 ppm)	5% Ag/TiO_2_ nanofibers (−)	Sol-gel + electrospinning	75 W Xe arc lamp	70	240	[54]
Acetaldehyde gas (100 ppm_v_)	0.5%Ag/TiO_2_ nanofibers (0.05 g)	Electrospinning and post-thermal process	UV light	98.4	140	[55]
Methyl orange and ethanol (1.0 ppm)	Cellulose/Ag-doped TiO_2_ nanofibers	TEMPO-mediated oxidation method	UV-B illumination (8.0 W)	88	720	[56]
4-nitrophenol (4-NP) (0.1 mM)	Ag/TiO_2_/PVDF@TiO_2_ nanofibers	Epitaxial growth	Membrane filtration	97	30	[57]
Parathion (0.05 μg/mL)	4% Ag/TiO_2_ (0.2 g/L)	Calcination of electrospun PVP/Ti(OiPr)_4_/AgNO_3_	UV light (λ ≥ 254 nm, 6 W)	90	60	[58]
Phenol 100 mg L−1	1%Ag–TiO_2_–SiO_2_ (1 g/L)	Sol–gel method	500-W xenon lamp	91	180	[59]
MB (10 ppm)	Ag/TiO_2_ nanofiber membrane	Polyol synthesis	(Xenon arc lamp, 100 mW/cm^2^	80	30	[60]
Glucose	2 wt % Ag–TiO_2_	*In situ* method calcined under N_2_	UV-A	99	120	[61]
MB (3 ppm)	Plasma-PVA/Ag–TiO_2_	Electrospinning + plasma treatment	Visible light 3 W/m^2^	51	360	[62]
Rhodamine-B (10 ppm)	Ag-coated TiO_2_ 1 g/L	Electrospinning + silver mirror reaction	Xe lamp 300W	45	360	[63]
MB (10 ppm)	Ag–TiO_2_ (0.4 g/L)	Electrospinning + sol-gel	Halogen lamp 400W	97	240	[64]

To further understand the kinetics of ACT degradation under visible light, we modeled it using a pseudo-first order reaction due to the linearity of the curve, with the linear coefficient R^2^ approaching 1 (Fig. 6c). The degradation rate of ACT in AgHBN surpassed that of the Ag-0.5, Ag-1.5, and Ag-3 samples owing to the establishment of a heterojunction within the nanocomposite involving BN. The highest rate constant, 0.03458 min^−1^, was achieved with AgHBN, which was 6.70 and 3.75 times higher than those of H95T5 and Ag-3, respectively.

Subsequently, we quantified ACT degradation over five cycles. After each cycle, the AgHBN catalyst underwent filtration, washing in water, and drying at 100 °C, as previously described [32,33,42]. The degradation of ACT exhibited a gradual decline, decreasing from 99.29% in the initial cycle to 97.30% in the second cycle, 96.31% in the third cycle, 95.31% in the fourth cycle, and 93.40% in the fifth cycle, as illustrated in Fig. 6d. The ∼6% loss of catalytic activity after five cycles indicated that doping with BN nanosheets improves the catalyst stability.

In conclusion, our research has led to the development of an innovative photocatalyst that excels in efficiently removing dyes and pharmaceuticals from wastewater and demonstrates remarkable stability over multiple cycles. The innovative Ag-BN/HNT-TiO_2_ nanofibers, predominantly made from natural halloysite nanotubes obtained from Tunisia, display significant potential as a framework for photocatalysis. The integration of Ag nanoparticles and BN nanosheets plays a crucial role in boosting their performance. These findings underscore the significant potential of this hybrid nanofiber composite in addressing environmental remediation challenges with an environmentally friendly and cost-effective approach. Our work opens the door to practical applications in the field of wastewater treatment, offering a sustainable solution for addressing water pollution issues and promoting a cleaner and healthier environment.

**Fig. 7:**
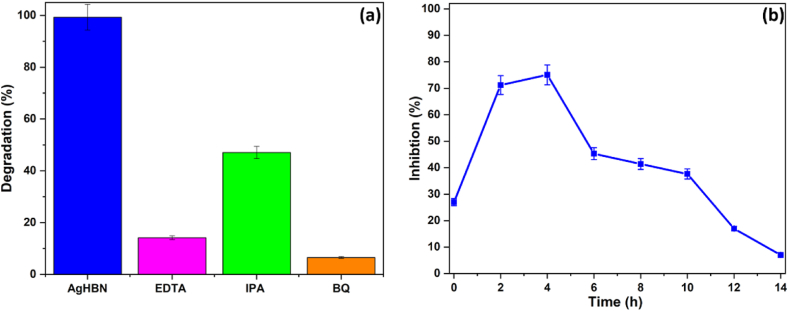
ACT degradation facilitated by AgHBN alongside EDTA, IPA, or BQ (a), Inhibition of V. fischeri fluorescence during ACT degradation using AgHBN under visible light exposure (b).

Next, ACT degradation was carried out in the same experimental conditions, but in the presence of AgHBN alone and also of different scavengers (0.06 M/each [32]) (Fig. 7a). The introduction of EDTA (positive hole scavenger), isopropanol (hydroxyl radical scavenger), and *p*-benzoquinone (superoxide anion scavenger) resulted in respective reductions of 85.7%, 52.5%, and 93.4% in the degradation rate of ACT. This suggests that all three radicals play a role in ACT degradation under visible light exposure [33,42].

To explore the generation of harmful by-products during ACT degradation, a study was conducted by exposing *V. fischeri* to the AgHBN and ACT solution. Fluorescence intensity of V. fischeri was measured at various time intervals during visible light exposure. *V. fischeri* fluorescence was inhibited by 27% after 15 min and by 71% after 2 h (Fig. 7b). Then, the inhibition rate decreased: 45% at 6 h, 41% at 8 h, 17% at 12 h and 7% at 14 h. Therefore, after 14 h of photodegradation, the toxic aromatic by-products were transformed into non-toxic compounds [33,42].

**Fig. 8:**
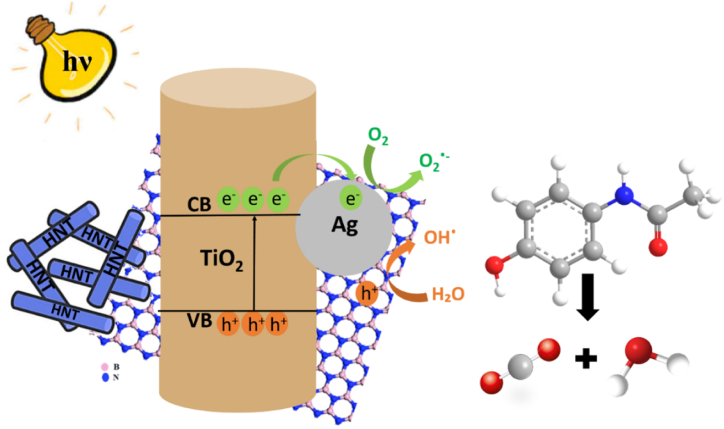
Visualizing the efficiency of AgHBN catalyst in decomposing ACT using visible light.

Fig. 8 depicts a diagram illustrating the mechanism by which ACT degradation occurs in the presence of the AgHBN catalyst. Under visible light exposure, this catalyst facilitates the creation of electron-hole pairs. These pairs interact with water and oxygen molecules adhered to the AgHBN surface, resulting in the formation of hydroxyl (^•^OH) and superoxide anion (O_2_^•−^) radicals. Additionally, Ag plays a role in the movement of photo-induced electrons from the TiO_2_ valence band towards an acceptor, thereby aiding in the generation of superoxide radicals. Furthermore, electrons become trapped by oxygen vacancies and the extensive Ag surface area on TiO_2_ surface, effectively preventing electron-hole (e^−^/h^+^) recombination [65].

Moreover, in AgHBN, the presence of B–O–Ti bonds disrupts the charge balance caused by oxygen holes, influencing the capture of photogenerated electrons. These electrons can be transferred to the π–π conjugate system of BN, inhibiting electron-hole pair recombination [33]. Consequently, this leads to an enhancement in AgHBN’s photocatalytic efficiency, ultimately contributing to the complete breakdown of ACT into carbon dioxide and water.

## 4 Conclusion

Ag/HNT-TiO_2_ and BN-Ag/HNT-TiO_2_ (AgHBN) composite nanofibers were successfully synthesized through electrospinning. Various characterization techniques, including SEM, TEM, EDX, Raman spectroscopy, and XRD, confirmed the incorporation of Ag and BN nanosheets. The introduction of Ag resulted in a downward shift in TiO_2_ diffraction peaks, while the addition of BN led to an upward shift, showing the emergence of new stages characterized by altered lattice dimensions. BET measurements demonstrated the creation of new active adsorption sites upon the inclusion of Ag nanoparticles and BN nanosheets. XPS examination revealed the existence of both Ag and BN elements. The photocatalytic activity of Ag/HNT-TiO_2_ and AgHBN was significantly enhanced, with ACT degradation rates two to three times faster, respectively, compared to H95T5 nanofibers. This enhancement was attributed to efficient charge separation, as photo-generated holes transferred from TiO_2_ to BN nanosheets. AgHBN exhibited sustained stability over five cycles. The photodegradation process was predominantly catalyzed by ^•^OH, h^+^, and O_2_^•−^ radicals, as confirmed by scavenger tests. Although toxic by-products were formed during the degradation process, they were subsequently transformed into non-toxic compounds, as demonstrated by *V. fischeri*-based toxicity tests. In conclusion, the AgHBN nanocomposite, comprising 5% TiO_2_, 3% Ag, and 5% BN, demonstrates promising photocatalytic efficiency and long-term stability. It stands as a cost-effective candidate for large-scale production of a photocatalyst for water pollutant removal.

## Data availability statement

Authors will provide data upon reasonable request.

## CRediT authorship contribution statement

**Mahmoud Abid:** Writing – original draft, Validation, Methodology, Formal analysis, Data curation, Conceptualization. **Igor Iatsunskyi:** Writing – original draft, Resources, Funding acquisition, Formal analysis, Data curation. **Emerson Coy:** Writing – original draft, Resources, Funding acquisition, Data curation, Conceptualization. **Geoffroy Lesage:** Writing – review & editing, Validation, Supervision, Resources, Funding acquisition, Formal analysis. **Abdesslem Ben Haj Amara:** Writing – review & editing, Validation, Supervision, Resources, Funding acquisition. **Mikhael Bechelany:** Writing – review & editing, Validation, Supervision, Resources, Project administration, Methodology, Funding acquisition, Conceptualization.

## Declaration of competing interest

The authors declare that they have no known competing financial interests or personal relationships that could have appeared to influence the work reported in this paper.
